# T cell-activation in neuromyelitis optica lesions plays a role in their formation

**DOI:** 10.1186/2051-5960-1-85

**Published:** 2013-12-24

**Authors:** Maria Pohl, Naoto Kawakami, Maja Kitic, Jan Bauer, Rui Martins, Marie-Therese Fischer, Joana Machado-Santos, Simone Mader, Joachim W Ellwart, Tatsuro Misu, Kazuo Fujihara, Hartmut Wekerle, Markus Reindl, Hans Lassmann, Monika Bradl

**Affiliations:** 1 Department of Neuroimmunology, Center for Brain Research, Medical University Vienna, Spitalgasse 4, Vienna A-1090, Austria; 2 Max-Planck-Insitute for Neurobiology, Max-Lebsche-Platz 31, Munich 81377, Germany; 3 Institute of Clinical Neuroimmunology, LMU Munich, Max-Lebsche Platz 31, Munich 81377, Germany; 4 Clinical Department of Neurology, Innsbruck Medical University, Innrain 66/2, Innsbruck A-6020, Austria; 5 Helmholtz Center Munich, Institute of Molecular Immunology, Marchioninistr. 25, Munich 81377, Germany; 6 Departments of Multiple Sclerosis Therapeutics and Neurology, Tohoku University Graduate School of Medicine, 1-1 Seiryomachi, Aobaku, Sendai 980-8574, Japan

**Keywords:** Neuromyelitis optica, T cell activation, Aquaporin 4, Lesion, IFN-γ

## Abstract

**Background:**

Neuromyelitis optica (NMO) is an inflammatory demyelinating disease of the central nervous system (CNS), which is characterized by the presence of pathogenic serum autoantibodies against aquaporin 4 (AQP4) in the vast majority of patients. The contribution of T cells to the formation of astrocyte destructive lesions is currently unclear. However, active human NMO lesions contain CD4^+^ T-lymphocytes expressing the activation marker Ox40, and the expression is more profound compared to that seen in MS lesions of comparable activity. Therefore, we analyzed the role of T-cell activation within the CNS in the initiation of NMO lesions in an experimental model of co-transfer of different encephalitogenic T-cells and human AQP4 antibody containing NMO immunoglobulin (NMO IgG). We further studied the expression of the T-cell activation marker Ox40 in NMO and multiple sclerosis lesions in different stages of activity.

**Results:**

All encephalitogenic T-cell lines used in our experiments induced brain inflammation with a comparable extent of blood brain barrier damage, allowing human NMO IgG to penetrate into the brain and spinal cord tissue. However, astrocyte destructive NMO lesions were only seen with T-cells, which showed signs of activation in the lesions. T-cell activation was reflected by the expression of the activation marker Ox40 and pronounced production of γ-IFN, which was able to increase the production of complement proteins and of the Fc gamma III receptor (Fcgr3) and decreased production of complement inhibitory protein Factor H in microglia.

**Conclusions:**

Our data indicate that local activation of T-cells provide an inflammatory environment in the CNS, which allows AQP4 auto-antibodies to induce astrocyte destructive NMO-like lesions.

## Background

Neuromyelitis optica (NMO) is an astrocytopathic disease of the central nervous system (CNS) characterized by optic neuritis, transverse myelitis, and – in the vast majority of patients – by the presence of specific auto-antibodies, the so-called NMO-IgGs [1]. These antibodies are directed against aquaporin 4 (AQP4), a water channel particularly enriched on astrocytic processes at the glia limitans [2,3], and they are pathogenic. They can bind to AQP4 on the surface of astrocytes, fix complement, and initiate complement-mediated destruction of these cells [4,5]. Based on earlier findings in NMO models it became clear that the presence of serum NMO-IgG is not sufficient to initiate astrocyte-destructive lesions [5], similar to the situation in NMO patients, who can be NMO-IgG seropositive for many years without showing clinical evidence of NMO [6]. Interestingly, even the presence of NMO-IgG and complement components in the CNS parenchyma is insufficient to promote the formation of large experimental NMO lesions, as seen when NMO-IgG and complement is injected directly into the striatum of mice with low numbers or abolished effector functions of circulating neutrophils [7], or when NMO-IgG and complement gain access to the brain through a blood–brain barrier that is developmentally leaky [5,8] or was rendered leaky due to the action of toxins [8] or to the intra-cerebral injection of cytokines [9]. All these observations clearly suggested that additional effector mechanisms are needed to initiate/promote astrocyte-destructive lesions. Further studies revealed that these mechanisms are triggered by CNS inflammation. In NMO patients, active NMO lesions are characterized by complement deposition on and subsequent destruction of astrocytes, and have a pronounced inflammatory component as evidenced by the presence of T cells, many neutrophils, macrophages/activated microglia cells and some eosinophils [10,11]. In experimental NMO, lesions were induced by myelin basic protein (MBP)-specific T cells in NMO-IgG seropositive Lewis rats, and faithfully reproduced essential features of active lesions in NMO patients [4,5]. Since the antigen recognition of T cells found in NMO lesions is still unknown, the findings in experimental NMO raise important questions: Are all CNS antigen-specific T cells similarly able to initiate astrocyte-destructive lesions in NMO-IgG seropositive hosts? And if not, what are the requirements for T cells to do so? These questions were answered in the current study.

## Methods

### NMO IgG preparation

The IgG preparation of the NMO patient J0 used has been extensively characterized before [5], and is termed NMO-IgG throughout this article. Its use was approved by the Ethics Committee of Tohoku University School of Medicine (No. 2007–327).

### Human tissue samples

Autopsy CNS tissue of 6 NMO and 10 MS patients (Table 1) from paraffin blocks and sections archived in the Center of Brain Research, Medical University Vienna, Austria or in Tohoku Medical University was used. The study was approved by the Ethical Committee of the Medical University of Vienna (EK. No. 535/2004 and 087/01/2012).

**Table 1:** Characteristics of the NMO and MS tissues used

**Disease**	**Activity**	**Region**
**case-block**		
NMO 1-1	LA	Cerebellum, medulla oblongata
NMO 1-2	EA	Spinal cord
NMO 1-3	LA	Spinal cord
NMO 1-4	EA	Spinal cord
NMO 1-5	LA	Mesencephalon
NMO 1-6A	LA	Periventricular (4th ventricle)
NMO 1-10A	LA	Temporal lobe
NMO 2-49	Acute, EA	Optic nerve
NMO 3-2	Chronic, IA	Spinal cord
NMO 4-49	Subacute/chronic LA-IA	Spinal cord
NMO 5-2	EA, LA	Medulla oblongata
NMO 6-2	LA	Spinal cord
NMO 6-4	IA	Medulla oblongata
AMS 1-1A	EA, LA, IA	Spinal cord
AMS 1-10D	EA, LA, IA	Temporal lobe
SPMS 1-12	SEL, IA	Optic nerve
SPMS 1-10	SEL IA	Medulla
SPMS 2-19	SEL IA	Spinal cord
SPMS 2-20	SEL, IA	Spinal cord
SPMS 2-21A	SEL, IA	Spinal cord
AMS 6-15	IA	Spinal cord
SPMS 3-31	SEL, IA	Medulla oblongata
AMS 2-6	EA	Brain
RRMS 1	EA, LA	Brain
AMS 3-5	EA, LA	Brain
AMS 4-2	EA, LA	Brain
AMS 5-97	EA, LA	Brain

Active lesions were identified and staged based on their dense infiltration by macrophages/activated microglia, and, in the case of NMO, by the presence of large numbers of neutrophilic granulocytes. Macrophages contained either early myelin degradation products (i.e. they were MOG+; early active lesions (EA)) or late myelin degradation products (i.e. they were PLP+; late active lesions (LA)). Inactive lesions (IA) had sharp lesion borders without macrophage infiltration of microglia activation, and slowly expanding lesions (SEL) were classified according to their inactive center, surrounded by a rim of activated microglia with some macrophages with myelin degradation products at the lesion margin.

### Animals

8 week-old Lewis rats from Charles River Wiga (Sulzfeld, Germany) were used. They were housed in the Decentral Facilities of the Institute for Biomedical Research (Medical University Vienna) under standardized conditions. The experiments were approved by the Ethics Committee of the Medical University Vienna and performed with the license of the Austrian Ministery for Science and Research.

### Induction of experimental autoimmune encephalomyelitis (EAE) and tissue preparation

T cell lines against myelin basic protein (MBP, from guinea pig, Sigma), myelin oligodendrocyte glycoprotein (MOG, recombinant N-terminal peptide 1–125, rat, own production) and the astrocytic Ca^2+^ binding protein S100β (bovine, Sigma) were intraperitoneally injected to induce EAE. These T cell lines have been established and expanded under conditions favoring TH1 cells, but not TH17 cells. Weight loss as earliest clinical sign of EAE started 4 days after the transfer. At this time point the animals received an intra-peritoneal injection with 1 ml phosphate buffered saline (PBS) containing either 10 mg NMO-IgG [5] or 10 mg normal human IgG (Subcuvia^R^) [5]. 24 hours later, the animals were sacrificed with CO_2_ and perfused with 4% phosphate buffered paraformaldehyde (PFA). Brains and spinal cords were dissected, immersed for another 18 hours in PFA, and embedded in paraffin. The production of T cells transduced with green fluorescent protein (GFP), EAE induction with these cells, their re-isolation from CNS lesions and their characterization by flow cytometry followed established procedures [12,13].

### Determination of IFN-γ production by polymerase chain reaction

RNA was purified using the RNeasy kit with QIAshredders (Qiagen), converted to cDNA, and subjected to real-time polymerase chain reactions (RT-PCR) using the Power SYBR® Green PCR Master Mix in a StepOnePlus RT-PCR system (both from Applied Biosystems) according to the manufacturers’ instructions. A 25 μl reaction mixture was used for each sample, containing cDNA and primer sets (200 nM) for IFN-γ (forward: 5′-ATTCATGAGCATCGCCAAGTTC-3′, reverse:

5′-TGACAGCTGGTGAATCACTCTGAT-3′; available at Real-Time Primer and Probe Database, RTPrimerDB ID:3773; https://medgen.ugent.be/rtprimerdb/; [14]) or beta actin (Actb; forward: 5′-AGGCCAACCGTGAAAAGATG-3′; reverse: 5′-ACCAGAGGCATACAGGGACAA-3′; [15]). The initial 10-min denaturation step at 95°C was followed by 40 cycles of denaturation (95°C, 15 s) and annealing/extension (60°C, 1 min). The absence of non-specific amplification was determined by melt curve analysis. All reactions were run in duplicates.

### Immunohistochemistry

All tissue blocks were cut and stained as described [5], using the following antibodies: W3/13 (T-cells and granulocytes; mouse monoclonal; Serotec, UK); ED1 (macrophages, microglia; mouse monoclonal; Serotec, UK); commercial anti-AQP4 (rabbit polyclonal; Sigma, Germany); anti-GFAP (rabbit polyclonal; Dako, Denmark, or mouse monoclonal; Neomarkers, USA); anti-human immunoglobulin (biotinylated donkey; polyclonal; Amersham, UK) and anti-complement C9 (rabbit polyclonal [16]). Immunohistochemistry was completed by using appropriate biotinylated secondary antibodies (sheep anti-mouse, donkey anti-rabbit, donkey anti-sheep/goat; all from GE Healthcare/Amersham) and subsequent incubation of the sections with peroxidase-labelled avidin (Sigma, Germany).

For double immunostainings of OX40 and T cell markers, sections were incubated with anti-OX40 (1:30, Pharmingen) overnight at 4°C, washed, further reacted with biotinylated donkey-anti-rabbit antibodies (1:500 Jackson ImmunoResearch, West Grove PA, USA) for 1 hour at RT, incubated with avidin-alkaline phosphatase (Sigma, Germany) and developed with Fast Blue (FB, Sigma, Germany) substrate. To retrieve CD4 or CD8 epitopes and inactivate the binding properties of the first round of antibodies (anti-Ox40 and biotinylated anti-rabbit), sections were steamed in a food steaming device in TRIS buffer containing EDTA (10 mM, pH = 9.0) for 90 minutes. Then, anti-CD4 (Ab8, Neomarkers, 1:100 plus 1 F6, Acris, 1:500) or anti-CD8 antibodies (SP16, Neomarkers, 1:250) were applied overnight at 4°C, followed by washing and incubation with biotinylated donkey-anti-rabbit antibodies and tyramide enhancement [17]. Finally, avidin-peroxidase (Sigma) was applied and sections were developed with aminoethyl carbazole (AEC, Sigma).

For double immunostainings of proliferating cell nuclear antigen (PCNA) and OX40, sections were steamed in citrate buffer for 30 minutes. Then, mouse anti-PCNA (clone PC10, Dako, 1:50000) was applied overnight at 4°C. The sections were washed, incubated with biotinylated anti-mouse antibodies (Jackson ImmunoResearch, West Grove PA, USA, 1:500) for 1 hour, washed again, incubated with avidin-alkaline phosphatase (Sigma, Germany) and developed with Fast Blue (FB, Sigma, Germany) substrate. Afterwards, the sections were steamed for 45 minutes with TRIS buffer containing EDTA (10 mM, pH = 9.0) to destroy free binding sites of the biotinylated anti-mouse antibodies used before, incubated with anti-OX40 (1:1000) overnight at 4°C, washed, further reacted with biotinylated anti-mouse antibodies (1:500) for 1 hour at RT and tyramide enhancement [17]. Finally, avidin-peroxidase (Sigma) was applied and sections were developed with aminoethyl carbazole (AEC, Sigma).

For fluorescent double labeling, Ox40 (mouse antibody) and anti-CD3 (rabbit antibody) were applied simultaneously at 4°C overnight. After washing with PBS, goat-anti-rabbit Cy3 (Jackson ImmunoResearch, West Grove, Philadelphia; 1:200) and biotinylated anti-mouse (Amersham Pharmacia Biotech; 1:200) antibodies were applied simultaneously for 1 hour at RT. The staining was finished by application of streptavidin-Cy2 (Jackson ImmunoResearch; 1:75) for 1 hour at RT. Fluorescent preparations were examined using a confocal laser scan microscope (Leica SP5, Leica Mannheim, Germany). Scanning for Cy2 (488 nm) und Cy3 (543 nm) was performed sequentially to rule out fluorescence bleed-through.

### Microglia cultures and gene expression profiling upon IFN-γ-treatment

Microglia cultures were essentially produced as described [18] and had a purity of ~99%. They were treated with 100 ng/ml IFN-γ for 48 hrs. Then, total RNA was isolated, the mRNA transcribed to cDNA and sent to ImaGenes (Berlin, Germany) for gene expression studies, using 4x44 K Multiplex whole rat genome microarrays (Agilent G4131F). The raw data were subjected to quantile normalizations prior to comparison between groups and calculation of fold changes in expression.

### Statistical evaluation

Statistical evaluations were performed using the PASW statistics 18 software system (SPSS Inc., Chicago, USA).

## Results

### Active NMO lesions contain activated CD4^+^ T cells

**Figure 1:**
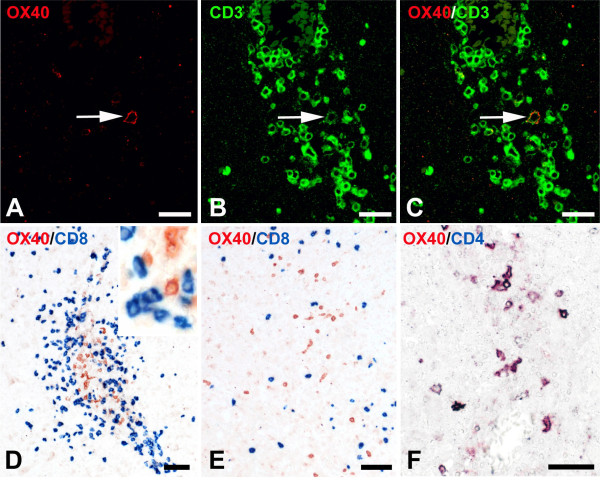
**Ox40 expression by T cells in NMO lesions. (A-C)** Confocal microscopy of a perivascular early active NMO lesion stained with antibodies against Ox40 (**A**, red) and CD3 (**B**, green) (overlay **C**, yellow). The Ox40 antigen is expressed by a subset of T cells (white arrow). **(D-F)**: Further stainings of NMO lesions with antibodies against Ox40 (brown) and CD8 (blue) **(D-E)** or CD4 (blue, **F**) reveals a complete absence of Ox40 expression on CD8^+^ T cells, both in perivascular lesions **(D)** and in the parenchyma at the edge of a demyelinating lesion **(E)**. In contrast, Ox40 products are expressed by CD4^+^ T cells (purple), as seen in this perivascular lesion. Bars: 25 μm **(A-C)** and 50 μm **(D-F)**.

**Figure 2:**
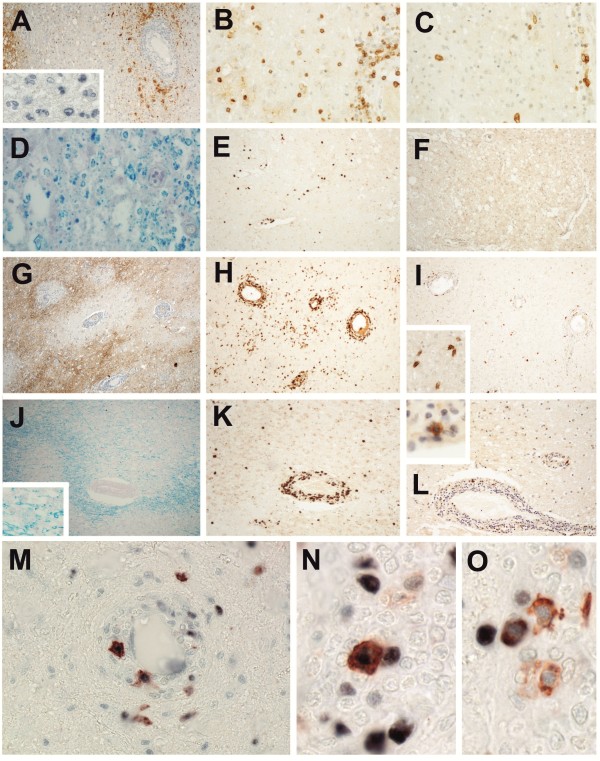
**Differences in Ox40 expression by CD4**^**+ **^**T cells between NMO and MS lesions.** Inflammatory lesions derived from spinal cords of NMO **(A,B,C)** and MS patients **(D,E,F)**, and from brains of NMO **(G,H,I)** and MS patients **(J,K,L)** were reacted with antibodies specific for AQP4 (brown reaction product; counterstaining with hematoxylin reveals nuclei in blue; **A**,**G**), CD3 (brown reaction product; **B**,**E**,**H**,**K**), Ox40 (brown reaction product; **C**,**F**,**I**,**L**) or were stained with Kluever-Barrera to reveal myelin (blue; **D**,**J**). The spinal cord lesions shown are early active, as revealed by the large number of granulocytes in NMO (inlay in **A**) and by the presence of myelin degradation products in macrophages in MS **(D)**. The brain lesions shown are late active in NMO, and early active, as revealed by macrophages containing myelin degradation products in MS (inlay in **J**). The inlays in I and L show Ox40^+^ T cells. In early active NMO lesions, 18% of all perivascular and 24% of all parenchymal OX40^+^ cells also express the proliferating cell nuclear antigen PCNA (**M**-**O**; PCNA visualized by the dark blue, Ox 40 by the brown reaction product).

**Figure 3:**
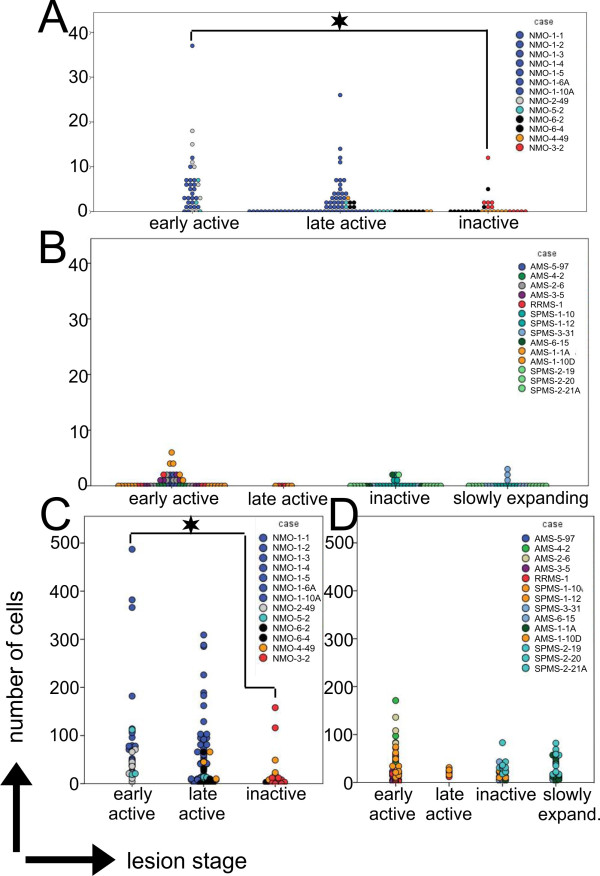
**Numbers of Ox40**^**+ **^**and CD3**^**+ **^**T cells in NMO and MS lesions at different lesion stages.** The number of Ox40 **(A,B)** and CD3 **(C,D)** positive T cells of 7 NMO **(A,C)** and 9 MS **(B,D)** cases was determined by evaluating regions of interest in NMO (ROI n = 144) and MS (ROI n = 112) (each region = 390.000 μm^2^) in spinal cord and brain lesions/inflamed parenchyma. Asterisks indicate statistically significant (p < 0,05; Mann Whitney U test with asymptotic significance (2 tailed)) differences between early active (n = 35) and inactive (n = 29) NMO lesions in the numbers of Ox40^+^ T cells **(A)** and CD3^+^ T cells **(C)**. The differences between early active (n = 51) and inactive (n = 34) MS lesions in the numbers of Ox40^+^ T cells **(B)** and CD3^+^ T cells **(D)** were not significant (p = 0,57 and p = 0,107, respectively). Please note that the differences in numbers of Ox40^+^ T cells and CD3^+^ T cells between early active NMO (n = 35) and early active MS (n = 51) is also highly significant (p < 0,0001 and p = 0,000318, respectively).

CD4^+^ T cells expressing the activation markers OX40 and PCNA are found in NMO lesions, independent of their location within the neuraxis (Figures 1 and 2). The numbers of Ox40^+^ T cells were significantly higher in early active than in inactive NMO lesions (Figure 3), and in early active NMO compared to early active MS. Moreover, also the total numbers of T cells in early active NMO lesions were significantly higher than in inactive NMO lesions, while there was no statistically significant difference in T cell numbers between early active, inactive, or slowly expanding MS lesions (Figure 3). What is the relevance of this finding for the formation of astrocyte-destructive lesions in NMO?

### T cells with different CNS antigen-specificities are differentially activated within the CNS

**Figure 4:**
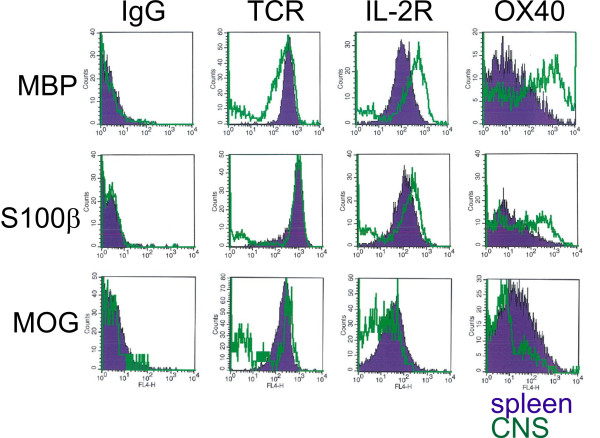
**T cells with different CNS antigen-specificity are activated to different extent in the CNS.** Analysis of surface markers by flow cytometry. Histograms are shown. GFP-labeled MBP-, S100β-, and MOG-specific T cells isolated from the spleen (blue) or spinal cord (green) of recipient rats were isolated at the acute phase of clinical symptoms and analyzed for the expression of T cell receptors (TCR), interleukin-2 receptors (IL-2R) or the Ox40 antigen, using specific antibodies for these molecules and an isotype control (IgG). MBP-specific T cells were strongly activated, as evidenced by a down-regulation of TCR, and an up-regulation of IL-2R and the Ox40 antigen. S100β-specific T cells showed an intermediated degree of activation (i.e. no downregulation of TCR, weak up-regulation of IL-2R and Ox40 antigen), and MOG-specific T cells were not noticeably activated, as revealed by the lack of upregulation of IL-2R and the Ox40 antigen).

To address this question, we studied the extent of T cell activation in EAE provoked by T cells with different CNS antigen-specificities. T cells specific for MBP, S100β, and MOG were labeled with GFP as described [12], and used for the induction of EAE. At the peak of EAE, we re-isolated these cells from the CNS, and studied their surface expression of the CD3-T cell receptor (TCR) complex, the IL-2R, and the Ox40 antigen as surrogate markers of T cell activation [12]. We found that MBP-specific T cells were strongly activated in the CNS, as indicated by a down-modulation of the TCR, and by increased levels of IL-2R and the Ox40 antigen. S100β-specific T cells had an intermediate activation status, as revealed by unchanged expression levels of the TCR, by marginally increased levels of IL-2R and moderately increased levels of the Ox40 antigen. MOG-specific T cells, finally, did not show any increase in IL-2R and Ox40 antigen expression, and were hence not significantly activated within the CNS (Figure 4, Additional file 1: Figure S1). This is in marked contrast to the situation in vitro, where all these cells can be readily activated and expanded in response to their cognate antigen in the context of rat MHC class II products ([12], own observation).

### T cells which are differently activated in the CNS differ in their ability to induce astrocyte-destructive lesions in NMO-IgG seropositive rats

**Figure 5:**
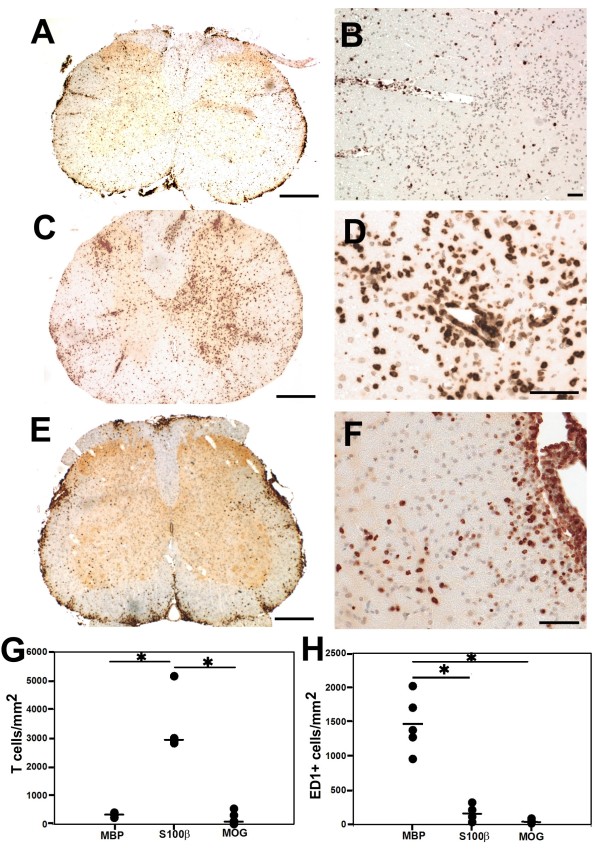
**T cells infiltration of the spinal cord following the initiation of NMO-like lesions in NMO-IgG seropositive animals by T cells with different CNS antigen-specificities. (A-F)** T cells specific for MBP **(A,B)**, S100β **(C,D)** and MOG **(E,F)** were used to induce CNS inflammation, followed by transfer of NMO-IgG 4 days later. The animals were sacrificed 5 days after T cell transfer. For histological evaluation, their spinal cords were reacted with anti-CD3 antibodies (brown reaction product) and counterstained with hematoxylin to reveal nuclei (blue). bars = 500 μm **(A,C,E)** and 100 μm **(B,D,F)**. **(G)** The average number of T cells per mm^2^ of lesions was determined by evaluating 5 representative spinal cord cross sections (1 cervical, 2 thoracal, 2 lumbar cross sections) per animal, using 5 animals (MBP- and MOG-specific T cells) or 4 animals (S100β-specific T cells) per group. Asterisks indicate statistically significant (p < 0,05) differences between individual CNS antigen specificities of the T cells used to induce CNS inflammation (Kruskal-Wallis followed by Mann–Whitney U test and Bonferroni-Holm correction; p = 0,0476 for MBP/S100β and S100β/MOG, p = 0.858 for MBP/MOG). **(H)** Numbers of ED1^+^ cells (activated microglia/macrophages) in spinal cord cross sections. The cell numbers were determined by evaluating one complete spinal cord cross section per animal, using 5 animals (MBP- and MOG-specific T cells) or 4 animals (S100β-specific T cells) per group. Asterisks indicate statistically significant (p < 0,05) differences between individual CNS antigen specificities of the T cells used to induce CNS inflammation (Kruskal-Wallis followed by Mann–Whitney U test and Bonferroni-Holm correction; p = 0,048 for MBP/S100β, p = 0,024 for MBP/MOG, and p = 0,189 for S100β/MOG).

**Figure 6:**
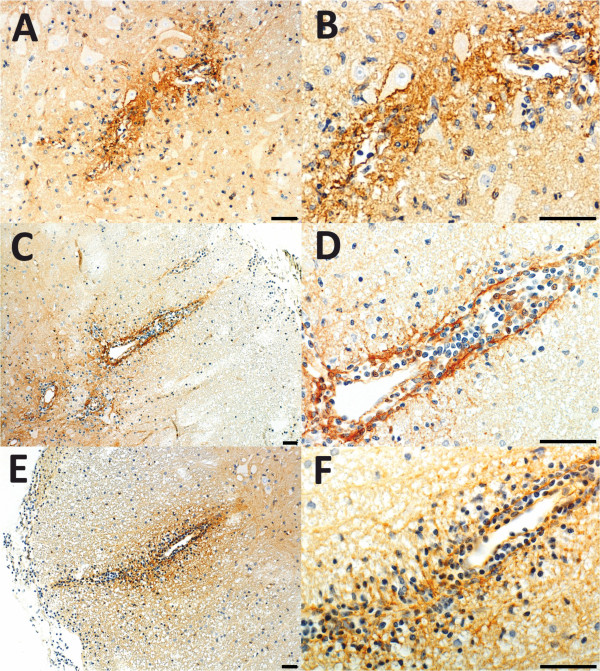
**Entry of human immunglobulins to lesions provoked by different CNS antigen-specific T cells in NMO-IgG seropositive animals. (A-F)** T cells specific for MBP **(A,B)**, S100β **(C,D)** and MOG **(E,F)** were used to induce CNS inflammation, followed by transfer of NMO-IgG 4 days after T cell transfer. The animals were sacrificed 5 days after T cell transfer. For histological evaluation, their spinal cords were reacted with anti-human IgG (brown reaction product) and counterstained with hematoxylin to reveal nuclei (blue). Overviews **(A,C,E)** and details **(B,D,F)** of representative spinal cord sections are shown. Bars = 100 μm.

**Figure 7:**
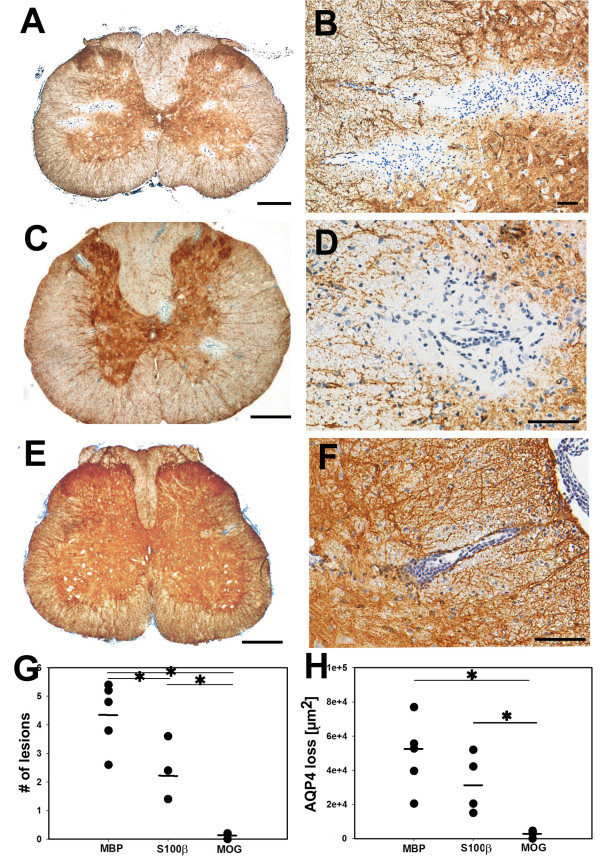
**Loss of AQP4 reactivity in NMO-like lesions initiated by T cells with different CNS antigen-specificities. (A-F)** T cells specific for MBP **(A,B)**, S100β **(C,D)** and MOG **(E,F)** were used to induce CNS inflammation, followed by transfer of NMO-IgG 4 days later. The animals were sacrificed 5 days after T cell transfer. For histological evaluation, their spinal cords were reacted with anti-AQP4 antibodies (brown reaction product) and counterstained with hematoxylin to reveal nuclei (blue). bars = 500 μm **(A,C,E)** and 100 μm **(B,D,F)**. **(G)** The average number of lesions with AQP4 loss per spinal cord cross section, as determined by evaluating 5 representative spinal cord cross sections (1 cervical, 2 thoracal, and 2 lumbar cross sections) per animal, using 5 animals (MBP, MOG) and 4 animals (S100β) per group. Asterisks indicate statistically significant differences between individual CNS antigen specificities of the T cells used to induce CNS inflammation (ANOVA-Holm Sidak; p < 0,001 for MBP-specific T cells compared to MOG-specific T cells; p = 0,008 for MBP-specific T cells compared to S100β-specific T cells; and p = 0,005 for S100β-specific T cells compared to MOG-specific T cells). **(H)** The largest lesion with AQP4 loss per animal, using 5 animals (MBP, MOG) and 4 animals (S100β) per group. Asterisks indicate statistically significant differences between individual CNS antigen specificities of the T cells used to induce CNS inflammation (Mann–Whitney U test with Bonferroni-Holm correction; p = 0,732 for MBP/S100β, p = 0,024 for MBP/MOG, p = 0,048 for S100β/MOG).

We then used T cells which are differently activated in the CNS, i.e. T cells specific for MBP-, S100β-, and MOG to induce EAE prior to the transfer of NMO-IgG. We found that all EAE models had similar NMO-IgG titres in the serum (between 1:320 and 1:1280; titres evenly distributed between the different groups),, that all T cells entered the CNS (Figure 5) and opened the blood–brain barrier for the entry of immunoglobulins (Figure 6). However, there were profound differences in the extent of astrocyte destruction. MBP-specific T cells induced the highest numbers of and the largest lesions with AQP4 loss (Figure 7). Also S100β-specific T cells facilitated the formation of astrocyte-destructive lesions, but the number of these lesions was significantly lower than the one found after transfer of MBP-specific T cells. In addition, there was a trend towards smaller lesions with AQP4-loss (Figure 7). Finally, MOG-specific T cells also infiltrated the CNS parenchyma, but did not induce astrocyte-destructive lesions (Figure 7). What are the differences between these T cells?

### Differences in T cell activation within the CNS translate into differences in the recruitment of macrophages/activated microglial cells

Differences in T cell activation have consequences for the expression of chemokines responsible for the recruitment of macrophages/activated microglial cells [12]. We therefore studied the numbers of these cells in the inflamed spinal cords of NMO-IgG seropositive hosts. We found that MBP-specific T cells recruited the highest numbers of activated microglial cells/macrophages, S100β-specific T cells intermediate numbers, and MOG-specific T cells the lowest numbers (medians 1464, 167, and 39 ED1^+^ cells/mm^2^ of spinal cord cross sections, respectively; Figure 5).

### Differences in T cell activation within the CNS translate into differences in IFN-γ production, which affects the microglial expression of complement factors/inhibitors and of Fcgr3

**Figure 8:**
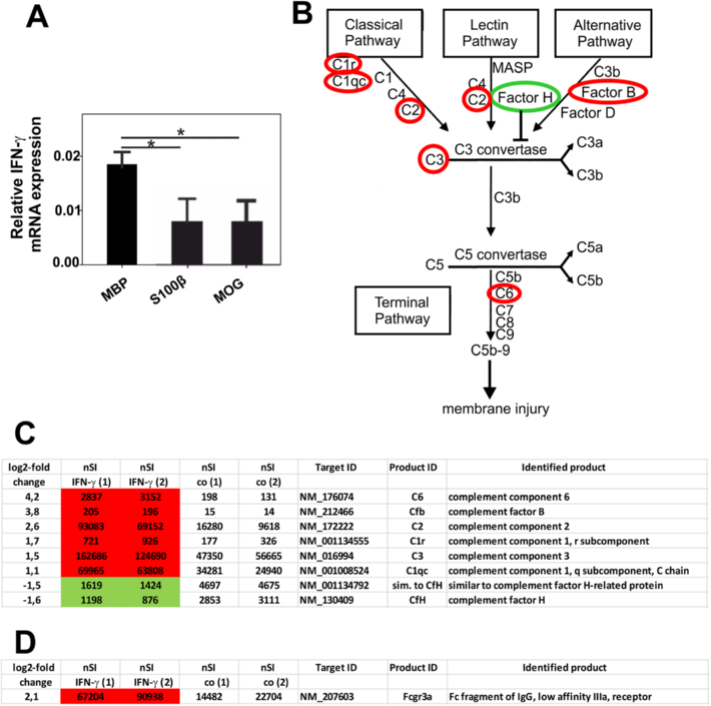
**Differences in T cell activation translate into differences in IFN-γ production, which affects the microglial expression of complement factors and complement inhibitors. (A)** Normalized relative expression of IFN-γ mRNA in relation to the house-keeping gene beta actin (calculated using the following equation: 2^-ΔCt^ = 2^-[Ct(GOI)-Ct(HKG)]^ (*GOI* – Gene of interest; *HKG* – house-keeping gene; [19]) are shown. Statistically significant differences (*, as determined by one-way ANOVA followed by Bonferroni’s post-hoc testing) were observed between MBP-specific T cells and their MOG- or S100β-specific counterparts. **(B)** Pathways contributing to the complement cascade [20] and alterations in gene expression (encircled in red: upregulation; encircled in green: downregulation) of complement factors and inhibitors by IFN-γ treated microglia. **(C-D)** Changes in gene expression of complement factors and inhibitors **(C)** and of Fcgr3 **(D)** in IFN-γ treated microglia. These cells were treated for 48 hrs with 100 ng/ml IFN-γ. Subsequently, the mRNA of these cells was harvested and subjected to gene expression profiling. Log2-fold changes in gene expression and differences in the normalized signal intensities (nSI) of complement components/factors and Fcgr3 between IFN-γ and vehicle control-treated microglial cultures are shown (2 different, independent samples per treatment group). Genes with elevated expression in the IFN-γ treated group are labeled red, genes with lower expression levels are labeled green.

Since EAE and NMO/EAE in Lewis rats are T_H_1-driven diseases with IFN-γ as lead cytokine, we next determined the IFN-γ expression of the re-isolated MBP-, S100β-, and MOG-specific T cells by RT-PCR, and observed that MBP-specific T cells had significantly higher relative IFN-γ mRNA expression levels than their S100β- or MOG-specific counterparts (Figure 8). We next stimulated microglia with IFN-γ or vehicle (control) *in vitro*, and studied the IFN-γ induced changes in the expression of transcripts encoding complement proteins and Fcgr3. We observed a profound upregulation of complement component 1, r subcomponent (C1r), complement component 1, q subcomponent, C chain (C1qc), complement component 2 (C2), complement component 3 (C3), complement component 6 (C6), and complement factor B (Cfb), coinciding with the downregulation of the inhibitory complement factor H (CfH) and of “similar to complement factor H-related protein” (sim. to CfH). We also found a profound upregulation of Fcgr3 (Figure 8).

## Discussion

Although T cells are regularly found in NMO lesions, our knowledge about these cells is rather limited. For example, we do not know yet whether these cells recognize AQP4 [21] or other CNS antigens, and whether these cells initiated the lesions or were non-specifically recruited to these sites. However, we learned from experimental models of MS or NMO that only migration-competent CNS antigen-specific T cells can cross through the brain capillary endothelium, open the blood–brain barrier for the subsequent entry of additional inflammatory mediators, and initiate inflammatory CNS lesions. This immigration phase is similar in all CNS antigen-specific T cells, irrespective of their pathogenicity [12]. However, only highly pathogenic T cells are subsequently activated within the CNS [12]. We show here that the extent of T cell activation within the tissue is a critical parameter for the formation of astrocyte-destructive lesions in NMO-IgG seropositive rats, and we provide evidence for the presence of activated CD4^+^ T cells in CNS lesions of NMO patients, suggesting that these lesions have been initiated by pathogenic T cells recognizing antigens in the CNS.

Full activation of T cells in the CNS – as exemplified in our study by MBP-specific T cells - translates into the profound up-regulation of Ox40, increased levels of IL-2R, down-modulation of TCR, and robust production of IFN-γ [12,22,23]. Engagement of Ox40 with its ligand supports the survival of T cells and augments antigen-driven TCR-signaling in these cells [24]. Interactions of Ox40 with Ox40L on the surface of antigen presenting cells enhances their production of pro-inflammatory cytokines like IL-1 beta or IL-6 and their surface expression of co-stimulatory molecules [25,26]. Interestingly, IFN-γ triggers the expression of Ox40L in microglia [27] and induces a microglial phenotype characterized by the up-regulation of complement factors and the down-regulation of complement inhibitors which could favor the antibody and complement mediated destruction of astrocytes in experimental NMO lesions. IFN-γ also induces the upregulation of Fcgr3, which plays an important role in antibody-dependent cell-mediated cytotoxicity (ADCC). The binding of the antibody Fc region to Fc gamma receptors on activated microglia/macrophages promotes their accumulation and plays an important role in NMO pathology, as evidenced by a reduction of tissue damage in NMO models involving mice lacking Fcgr3, mice treated with Fcgr blockers, or mice containing mutated NMO-IgG without ADCC function [28].

Fully activated T cells also produce CCL2 and CCL3 transcripts [12] which may further attract large numbers of macrophages/activated microglia to the lesions.

The intermediate activation of T cells – as exemplified by S100β-specific T cells – recruits much less macrophages/activated microglial cells, which is in line with lower expression levels of CCL2 and CCL3 in these cells than in their MBP-specific counterparts [12]. This activation stage may still provide enough surface expression of Ox40 to augment antigen-driven TCR-signaling, thus enabling low doses of antigen to promote responses normally only induced by higher doses [29]. Intermediately activated S100β-specific T cells only produce low amounts of IFN-γ, but may compensate for this by being present in the CNS parenchyma in high cell numbers. This could explain why S100β-specific T cells are still able to facilitate the formation of astrocyte-destructive lesions in NMO-IgG seropositive animals.

However, if T cells were not activated at all in the CNS – as exemplified by MOG-specific T cells –, recruited macrophages/activated microglial cells in extremely low numbers, and did not compensate for low IFN-γ production levels by high parenchymal cell numbers, astrocyte-destructive lesions in the presence of NMO-IgG and complement were not formed despite inflammation-induced blood–brain barrier leakage.

Is there any evidence for the activation of AQP4-specific T cells in NMO/EAE? This question could not yet be experimentally addressed, since all AQP4-specific rat T cells tested so far were only weakly encephalitogenic [22] and could not be re-isolated in sufficiently high numbers to test their surface expression of activation markers by FACS analysis, and since commercially available antibodies against the rat Ox40 antigen did not work in paraffin-embedded or frozen tissue. However, AQP4-specific T cells cause lesions with AQP4 loss in NMO-IgG seropositive hosts [22], which suggests an activation of these cells within the CNS.

It might be argued that NMO is a T_H_17-driven disease [30,31], while NMO/EAE is driven by T_H_1 T cells [5,21], and that our findings about the consequences of activated, IFN-γ producing cells might be irrelevant for NMO. However, human T_H_17 cells comprise a heterogeneous subset of cells including IFN-γ-producing cells [32-34] and have the ability to shift to T_H_1 cells [35].

It was unexpected to see significantly higher numbers of T-cells expressing the activation marker Ox40 in active NMO compared to active MS lesions. Since this was the case in early as well as late active lesions, and in brain as well as spinal cord lesions, this observation cannot simply be explained by stage or location dependent differences in the lesions. In NMO lesions, Ox40 expression is seen in CD4^+^ T cells, and it has been described that activated CD4^+^ T cells have prolonged Ox40 expression [24]. In MS lesions, however, the activated T cells might belong to the clonally expanded CD8^+^ T cell pool [36], and Ox40 expression in activated CD8^+^ T cells appears to be more transient compared to that in activated CD4^+^ T cells [24]. Hence, Ox40 expression in MS lesions may escape detection.

## Conclusion

In summary our data indicate that even in conditions of inflammation and blood–brain barrier damage alone, entry of AQP4 specific autoantibodies into the CNS is insufficient to trigger astrocyte destructive NMO lesions and that the additional activation of effector mechanisms in the lesions is necessary to facilitate complement-mediated and antibody-dependent cellular cytotoxicity. Such an environment is provided in T cell-mediated inflammation when invading T cells are antigen-specifically activated within the CNS. Our data further suggest that a similar mechanism may operate in human NMO lesions, since the infiltrating T-cells show the respective signs of activation. Whether these cells recognize AQP4 or other CNS antigens is not resolved yet, since CD4^+^ T cells specific for AQP4 [37,38] or PLP [37] have been observed in the peripheral blood of NMO patients.

## Competing interests

The authors declare that they have no competing interests.

## Authors‘ contributions

MP established T cell lines, performed the animal experiments and histologically analyzed all rat and human CNS tissues; NK established GFP-expressing T cell lines and characterized the re-isolated T cells by flow cytometry; JWE sorted the GFP-expressing T cells, JB established the OX40 stainings and made confocal microscopy, JM-S performed the PCNA studies, MK, RM and M-TF established microglia cultures, performed the treatments with IFN-γ and analyzed microglia by real-time PCR (MK) and with microarrays (RM, M-TF). SM determined the anti-AQP4 antibody titres of the experimental animals. TM and KF provided the NMO-IgG preparation J0. HW and MR provided intellectual input. MB and HL designed the study and planned all experiments. All authors read and approved the final manuscript.

## Supplementary Material

Additional file 1: Figure S1 T cells with different CNS antigen-specificity are activated to different extent in the CNS. Shown here are dotblots of an analysis of surface markers by flow cytometry. GFP-labeled MBP-, S100β-, and MOG-specific T cells isolated from the spleen or spinal cord of recipient rats were isolated at the acute phase of clinical symptoms and analyzed for the expression of T cell receptors (TCR), interleukin-2 receptors (IL-2R) or the Ox40 antigen, using specific antibodies for these molecules and an isotype control (IgG). MBP-specific T cells were strongly activated, as evidenced by a down-regulation of TCR, and an up-regulation of IL-2R and the Ox40 antigen. S100β-specific T cells showed an intermediated degree of activation (i.e. no downregulation of TCR, weak up-regulation of IL-2R and Ox40 antigen), and MOG-specific T cells were not noticeably activated, as revealed by the lack of upregulation of IL-2R and the Ox40 antigen). Click here for file
